# Childhood Lead‐Exposure Prevention Through Predictive Mapping of At‐Risk Housing Nationwide: A Free, Public‐Data Screening Map Checked Against Measured Blood Lead in 11 States

**DOI:** 10.1029/2026GH002053

**Published:** 2026-10-05

**Authors:** Eric C. Ritter

**Affiliations:** ^1^ Fluoro‐Spec Inc. (DetectLead.com) East Setauket NY USA

**Keywords:** childhood lead exposure, ecological risk model, American community survey, blood‐lead surveillance, geospatial screening, environmental justice

## Abstract

Childhood lead exposure causes irreversible neurodevelopmental harm, and no safe blood lead level in children has been identified. Measured childhood blood lead is collected unevenly across the United States, and many areas publish no neighborhood‐level surveillance, so the children at highest risk are often the hardest to find by measurement. Building on the U.S. Environmental Protection Agency finding that housing age and poverty predict where childhood lead exposure concentrates, we computed a lead‐exposure risk score for all 83,388 U.S. census tracts and 3,222 counties from public American Community Survey housing‐age and poverty data, and tested it against measured childhood blood lead in 11 states by Spearman rank correlation. On surveillance the source study never used, predicted risk tracked measured elevated blood lead at rho = 0.64 across Wisconsin's 1,308 census tracts, with five independent county‐scale states ranging from 0.48 to 0.77 and three further independent tract states (Massachusetts, Rhode Island, Minnesota) agreeing. A flexible model on the same public inputs explains only about half the tract‐level variance, so housing age and poverty leave most of the variation unexplained, and the map is a screening signal rather than a diagnosis. Every validation state is an older‐housing Northeast, Midwest, or Great Plains jurisdiction, so the low‐risk predictions for the newer‐built West and Sun Belt remain an untested extrapolation. Reproducible from public data and free to use, the map ranks where confirmatory testing should go first, including in states that publish no surveillance of their own.

## Plain Language Summary

Lead is poisonous to young children, and even low levels cause permanent harm to the developing brain. The surest protection is to find and fix lead hazards in a home before a child is exposed. Most lead hazards come from old paint, but the United States does not test every child or map every neighborhood, so families and health departments often cannot tell which homes are risky until a child has already been poisoned. We used free public data, the age of houses and the local poverty rate, to estimate which neighborhoods across the whole country are most likely to hold lead hazards. We then checked those estimates against real childhood blood‐lead testing in 11 states and found they agree at a moderate level: the neighborhoods our map ranks highest are where more children actually test high for lead. The map cannot tell any one family whether their home is safe, because only a physical test of the home can do that. What it can do is point limited testing and inspection toward the neighborhoods most likely to need them first, including the many states that publish no lead data of their own. It is free and open to anyone.

## Key Points

- A free risk map scores every United States census tract and county for childhood lead exposure using only public housing and poverty data
- Predicted risk agrees with measured childhood blood lead across 11 states the map was never fitted to, at a moderate rank correlation
- The map shows where confirmatory testing should go first, including in states that publish no neighborhood blood lead data of their own

## 1 Introduction

### 1.1 The Problem Has No Safe Threshold

Childhood lead exposure is a settled and quantified public‐health failure rather than an open question. The U.S. Centers for Disease Control and Prevention (CDC) reports no safe level of lead in a child's blood, and that even low levels can affect IQ, attention, and academic achievement (CDC, n.d.‐a). In 2021 the CDC lowered its blood lead reference value from 5.0 to 3.5 μg per deciliter, set at the 97.5th percentile of the blood‐lead distribution among U.S. children ages 1 to 5, so that more children with comparatively higher levels would be identified (CDC, 2021a). The agency notes that the reference value is not health‐based and not a regulatory standard. A result below it does not mean a child is unharmed (CDC, 2021a).

The aggregate damage is large and already incurred. As historical context, from exposure pathways now largely closed, McFarland et al. (2022), in the Proceedings of the National Academy of Sciences, estimate that childhood exposure to leaded gasoline cost the living U.S. population roughly 824 million cumulative IQ points, and that more than 170 million Americans alive in 2015, over half the population, had childhood blood‐lead levels above the clinical concern threshold of their era. The burden may persist for life: Brown et al. (2025), in a conference abstract in Alzheimer's & Dementia, link historical atmospheric lead at its 1960 to 1974 peak to higher odds of memory problems half a century later. The pathway has shifted, but it has not closed. Leaded paint in housing built before the 1978 residential ban remains a dominant present‐day source of childhood exposure, and the CDC has estimated that about 500,000 U.S. children ages 1 to 5 had blood‐lead levels at or above the older 5.0 μg‐per‐deciliter reference value, a count that is higher at the current 3.5 reference value (CDC, n.d.‐a).

### 1.2 The Surveillance Gap

Knowing that lead harms children at any dose is not the same as knowing which children to protect. The United States has no complete, population‐representative measurement of childhood blood lead. The CDC receives about 3 million blood‐lead test results per year, a fraction of the roughly 22 million children under six (CDC, n.d.‐b; CDC, n.d.‐d). But the selection matters more than the count. Testing is deliberately concentrated on children judged to be at higher risk, so the reported surveillance data are, in the CDC's own words, “not a population‐based estimate” and “not representative of a whole county or a whole state” (CDC, n.d.‐b). The agency points anyone seeking nationally representative prevalence to NHANES, a survey designed for estimation rather than for locating individual neighborhoods at risk (CDC, n.d.‐b). The result is a systematic blind spot. A child who is never tested, in a place where few children are tested, never appears in the data, and the places with the least testing are not randomly distributed.

The measured data that do exist are held by states and not the federal government, and access to them is uneven by jurisdiction. Some states publish blood‐lead surveillance through open data interfaces, and the CDC Environmental Public Health Tracking Network carries county‐level elevated‐blood‐lead measures for participating states (CDC, n.d.‐e). Others lock the same information in static reports or dashboards that require formal records requests to obtain at usable spatial resolution. In this patchwork, the resolution of the available evidence depends less on where the hazard is than on each state's data‐publishing posture. A family, clinician, or local health department in a low‐publishing state has no neighborhood‐level signal at all, even though the underlying housing and poverty drivers of risk are present and measurable there.

### 1.3 Why a Prediction‐First National Map Is Needed

When direct measurement is incomplete and unevenly accessible, the established response is to predict risk from variables that are measured everywhere. The U.S. Environmental Protection Agency (EPA) took exactly this approach. Zartarian et al. (2024), in Environmental Science and Technology, screened 73,086 census tracts containing at least one child under six in the 50 states and modeled lead‐exposure risk from publicly available indicators, mainly the age of housing and poverty, because surveillance and environmental‐data gaps make disproportionately exposed communities hard to find by measurement alone. They evaluated the predicted hotspots against approximately 1.9 million Michigan blood‐lead results (2006–2016) and approximately 2.3 million Ohio results (2005–2018), and found moderate‐to‐substantial agreement, Cohen's kappa 0.49 to 0.63. They also found that a reduced model built on three variables (the share of homes built before 1940, the share built before 1950, and the share of families with an income‐to‐poverty ratio above 2) predicted hotspots about as well as the full model, and they restate the premise that “there is no known level of lead exposure to be without risk” (Zartarian et al., 2024).

That EPA result establishes the method but does not deliver a usable public instrument, even though its inputs are open and national. The American Community Survey publishes year‐structure‐built down to the census‐tract level in table B25034 and poverty status in subject table S1701 (U.S. Census Bureau, 2023f, 2023d), so the same housing‐plus‐poverty risk signal can be computed for every tract in the country, including the states where no measured blood‐lead data are publicly accessible. A prediction‐first map turns an indicator that depends on each state's reporting choices into a uniform, neighborhood‐level warning that exists everywhere the Census reaches. The map does not diagnose any individual child and does not replace a blood test. It is a screening layer that shows where measured testing and on‐the‐ground hazard confirmation should go first.

To be explicit about what is and is not new here, the prediction method is not ours. Zartarian et al. (2024) established that housing age and poverty predict childhood lead exposure and validated it in Michigan and Ohio. We claim no new method and no new finding about lead. What this paper does is make that existing method usable where it currently is not. We compute it for every census tract and county in the country on the current Census vintage, the usable public surface their result implied but did not release. We test whether its predictions agree with measured childhood blood lead in nine states their study never used, which is the central evidence here, because agreement on data the method was not derived from is not guaranteed. And we release the code and the derived data so any reader can rebuild and re‐check the result. The point is not novelty. It is a working, checked, public tool for the many states that publish no blood‐lead data of their own. We treat the prior finding as a method to be re‐tested rather than as settled ground. The Michigan and Ohio comparisons reuse the source study's own surveillance and are therefore a consistency check, while Wisconsin, the five county states, and three further tract‐scale states (Massachusetts, Minnesota, and Rhode Island) are genuine out‐of‐sample tests.

### 1.4 Prior Work and Intellectual Lineage

The approach we describe here, predicting childhood lead‐exposure risk from publicly available housing and socioeconomic data, did not originate with us, nor with any single recent effort. It rests on three decades of epidemiology, survey science, geographic analysis, and applied machine learning carried out by public health departments, federal agencies, university research groups, and investigative journalists. We summarize that lineage below.

### 1.5 Early Geographic and Area‐Based Risk Models

Sargent and colleagues first put this on a quantitative footing. In a logistic analysis of 238,275 Massachusetts children, Sargent et al. (1995) found that the percentage of housing built before 1950, per‐capita income, the percentage of residents who were Black, and a poverty index were each independently associated with community lead‐poisoning rates. This is the direct methodological ancestor of nearly every housing‐age‐plus‐poverty index that followed.

A closely related census‐tract analysis of 17,956 children across Providence County, Rhode Island found that the share of houses built before 1950 carried the largest adjusted association with the proportion of children with elevated blood lead, with vacant housing also a strong predictor (Sargent et al., 1997).

As geographic information systems matured, several groups translated these statistical associations into operational screening and targeting tools. Reissman et al. (2001) demonstrated the use of GIS to link blood‐lead data and housing age in support of health‐department decisions about prevention activities. Roberts et al. (2003), working in Charleston County, South Carolina, geocoded tax‐assessor housing records and found that children in pre‐1950 housing were roughly 3.9 times as likely to have an elevated blood lead level as children in post‐1977 housing.

The most spatially ambitious strand of this work came from the Children's Environmental Health Initiative led by Marie Lynn Miranda. Miranda et al. (2002) combined blood‐lead screening, county tax‐assessor housing‐age data, and census data into GIS models intended to direct prevention programs. Kim et al. (2008) then resolved risk to the individual tax parcel across 18 North Carolina counties and offered a framework for replicating such models elsewhere, the finest‐grained housing‐age risk modeling in the literature before Flint.

Akkus and Ozdenerol (2014) later reviewed this body of GIS‐based work and treated the area‐based risk‐index tradition as a coherent subfield.

The Lead Exposure Risk Index developed by the Washington State Department of Health (first version, 2016) is where this research literature meets present‐day public tools. It scores each census tract by combining the age of the housing stock with the share of households at or below 125% of the federal poverty level. This index has become the most widely replicated operational formulation of the housing‐age‐plus‐poverty approach (Washington State Department of Health, Childhood Lead Poisoning Prevention Program, 2016).

### 1.6 National Lead‐Paint Surveys and the Housing‐Age Evidence Base

These models weight housing age heavily because national surveys establish how the probability of lead‐based paint varies by construction era. The original U.S. Environmental Protection Agency National Survey (1995) is the source of the canonical by‐era probabilities, on the order of 87% of units built before 1940, roughly 69% for 1940 to 1959, and roughly 24% for 1960 to 1977 (EPA, 1995).

Jacobs et al. (2002), reporting on the HUD National Survey of Lead and Allergens in Housing, estimated from a nationally representative sample that 38 million U.S. homes contained lead‐based paint and 24 million had significant lead‐based‐paint hazards, with markedly higher prevalence in the Northeast and Midwest.

The Department of Housing and Urban Development's American Healthy Homes Surveys updated these national estimates. The second survey, AHHS II, based on fieldwork in 2018–2019, estimated that roughly 34.6 million homes (about 29.4% of housing units) contain lead‐based paint and that about 21.9 million homes have dust‐lead hazards under the 2019 standard (U.S. Department of Housing and Urban Development, 2021).

### 1.7 Epidemiology of Low‐Level Lead Exposure

The urgency behind this prediction work comes from a separate body of epidemiology showing that lead harms children at progressively lower exposures. Needleman et al. (1979) established subclinical harm by relating dentine lead levels in deciduous teeth to deficits in cognitive and classroom performance.

Subsequent work pressed the harm below the long‐standing 10 μg/dL action level. Lanphear et al. (2000), using NHANES III data on 4,853 children, observed cognitive deficits at blood lead concentrations under 10 μg/dL, and Canfield et al. (2003) found, in a cohort of 172 children, an even steeper inverse relationship between blood lead and IQ within that low range.

The international pooled analysis by Lanphear et al. (2005), combining seven prospective cohorts totaling 1,333 children, found measurable intellectual deficits even among children whose blood lead never exceeded 7.5 μg/dL and reported no evidence of a threshold below which lead is safe. If harm has no floor, the case for finding at‐risk children before exposure rather than after is straightforward.

### 1.8 Public Mapping Efforts

Journalists and civic‐data teams brought area‐based risk estimation to a broad public audience, often by directly adopting the Washington State methodology. In 2016, Frostenson and Kliff at Vox published a national neighborhood map assigning each census tract a risk decile from housing age and poverty, with open‐source code, explicitly replicating the Washington State Department of Health approach (Frostenson & Kliff, 2016).

In the same year, Pell and Schneyer at Reuters compiled state blood‐lead surveillance into an interactive map and identified thousands of census tracts and ZIP areas with lead‐poisoning prevalence at least double that observed in Flint (Pell & Schneyer, 2016).

The same methodological lineage was subsequently institutionalized in two widely used data platforms. The City Health Dashboard, produced by NYU Langone Health, publishes a Lead Exposure Risk Index that bins housing units by construction era, weights them by lead likelihood, and combines them with the share of households at or below 125% of the federal poverty level, stating that its method was based on the Washington State Department of Health and Vox Media work (City Health Dashboard, n.d.). PolicyMap hosts a closely related “Risk of lead exposure” layer under an attribution to the same sources (PolicyMap, n.d.).

### 1.9 House‐Level and Address‐Level Machine Learning

A final strand moved from area‐based indices to predictive models resolved to the individual child, address, or parcel. Potash et al. (2015), a collaboration between the University of Chicago's Data Science for Social Good group and the Chicago Department of Public Health, built per‐child and per‐address risk models from historical blood‐lead results and building characteristics, the address‐level precedent later work built on.

Abernethy et al. (2018) extended parcel‐level prediction to lead service lines in Flint, Michigan, using property age, value, location, and city records, work that became the basis of the BlueConduit effort.

### 1.10 Relation to the Present Work and to Recent Federal Synthesis

The federal work of Zartarian and colleagues draws on all of these traditions and is the immediate basis for our paper. Their 2022 analysis catalogs and compares the prior housing‐age and sociodemographic indices (Zartarian et al., 2022), and their 2024 hotspots analysis applies a random‐forest model validated against several such indices (Zartarian et al., 2024).

This body of prior scholarship spans the founding area‐based regressions, the national survey infrastructure, the low‐level‐harm epidemiology, the public maps, and the house‐level machine‐learning models. We claim no new paradigm; this work is one further incremental step along a path many others laid down.

## 2 Methods

### 2.1 Overview and Design Rationale

We constructed a national, tract‐level index of predicted childhood lead‐exposure risk from two publicly documented determinants: the age of the housing stock and the prevalence of poverty. The choice of inputs is not novel. It follows the housing‐age‐plus‐poverty approach the Washington State Department of Health uses for its Lead Exposure Risk Index, which combines ACS 5‐year housing‐age and poverty measures into a single community‐level score (Washington State Department of Health, n.d.). The same two determinants anchor the indices the U.S. Environmental Protection Agency screened in its national hotspots analysis, where a reduced three‐variable random‐forest model (percent of homes built before 1940, percent built before 1950, and a family income‐to‐poverty measure) reproduced the hotspot pattern of the full five‐variable model (Zartarian et al., 2024). Lead‐based residential paint was banned for consumer use in 1978, so housing age is a direct proxy for leaded paint and the dust it generates; poverty proxies both deferred paint maintenance and reduced remediation capacity (CDC, n.d.‐f). We chose these inputs because both are published for every census tract on the same recent vintage. That uniform national coverage lets a single method reach states that publish no measured blood‐lead data.

Our contribution is not the index form but its national reconstruction on the most recent 5‐year American Community Survey (ACS) vintage, and its tract‐by‐tract validation against measured childhood blood lead, reported separately. The EPA hotspots analysis was built on 2010 Census geography and ACS 2013–2017 5‐year inputs (Zartarian et al., 2024). We rebuilt the index on ACS 2018–2022 5‐year estimates (U.S. Census Bureau, 2023c), which moves the housing and poverty measures forward roughly a decade and re‐bases the geography on 2020‐vintage census tracts.

### 2.2 Data Sources

All inputs are American Community Survey 2018–2022 5‐year estimates, the vintage released 7 December 2023 and current at the time of analysis (U.S. Census Bureau, 2023c). The 5‐year file is the only ACS product published down to the census‐tract level for the full universe of tracts. It is the correct vintage for small‐area estimates because the 1‐year file does not tabulate most tracts (U.S. Census Bureau, 2023c). We pulled four tables.

**Table:** 

Purpose	ACS table	Type	Key variables used
Housing age	**B25034**, Year Structure Built	Detail	B25034_001E (total units); B25034_011E (pre‐1940); B25034_010E, B25034_009E (1940–1959); B25034_008E, B25034_007E (1960–1979) (U.S. Census Bureau, 2023b)
Poverty	**S1701**, Poverty Status in the Past 12 Months	Subject	S1701_C01_001E (population for whom poverty status is determined), S1701_C03_001E (percent below poverty level) (U.S. Census Bureau, 2023d)
Total population	**B01003**, Total Population	Detail	B01003_001E (total population) (U.S. Census Bureau, 2023e)

B25034 reports occupied and vacant housing units by the decade built. The method uses its pre‐1940 category (B25034_011E) together with the 1940‐to‐1959 and 1960‐to‐1979 decade categories (B25034_007E through B25034_010E), which give the three construction‐era shares the housing‐age score requires (U.S. Census Bureau, 2023b). S1701 is the ACS subject table for poverty status. It publishes the percent‐below‐poverty estimate directly as S1701_C03_001E over the universe “population for whom poverty status is determined” (S1701_C01_001E), so no separate denominator is needed (U.S. Census Bureau, 2023d). B01003 supplies total population, used for population‐weighting in the residual analysis rather than for the risk score itself (U.S. Census Bureau, 2023e).

### 2.3 Census API Pipeline

Data were retrieved programmatically from the Census Data API. Detailed tables (B25034 and B01003) were requested from the ACS 2022 5‐year detailed‐tables endpoint and the subject table (S1701) from the parallel subject endpoint (U.S. Census Bureau, 2023c):

https://api.census.gov/data/2022/acs/acs5?get=NAME,group(B25034)&for=tract:*&in=state:{FIPS}&key={KEY}

https://api.census.gov/data/2022/acs/acs5/subject?get=NAME,group(S1701)&for=tract:*&in=state:{FIPS}&key={KEY}

The API caps tract‐level wildcard queries at one state per call. The pipeline therefore iterated in = state:{FIPS} across the 50 states and the District of Columbia for tracts (plus Puerto Rico for counties), then concatenated the responses. County‐level inputs were retrieved with for = county:*. Estimates were joined to 11‐digit tract GEOIDs (2‐digit state + 3‐digit county + 6‐digit tract) and to 5‐digit county GEOIDs. We dropped records before scoring if their housing‐unit denominator (B25034_001E) was missing, zero, or carried a Census suppression sentinel. Where an individual era‐band or poverty cell was itself missing or suppressed, it was set to zero rather than the record dropped; at the tract scale this affects only a small number of very low‐population tracts and none of the validation joins. This is what reduces the raw tract universe to the scored set described below. Margins of error are published for every estimate (the _M‐suffixed variables) and were retained but not propagated into the point score (U.S. Census Bureau, 2023b).

### 2.4 Index Construction

The score combines housing age and poverty, both from the American Community Survey 2018–2022 5‐year estimates. Housing age enters as three construction‐era shares, weighted so that older stock counts for more, because the probability that a home contains lead‐based paint rises sharply with age. We define the inputs, give the exact Census fields, then state the model. Each quantity is computed once per census tract and once per county.

- s40: the share of housing units built in 1939 or earlier, the oldest and highest‐lead stock.
- s4059: the share built between 1940 and 1959.
- s6079: the share built between 1960 and 1979, the last era before the 1978 residential lead‐paint ban.
- poverty: the share of people living below the federal poverty line.

**Table:** 

Input	ACS source table	Computation from published Census fields
s40	B25034 (Year Structure Built)	B25034_011E/B25034_001E
s4059	B25034 (Year Structure Built)	(B25034_009E + B25034_010E)/B25034_001E
s6079	B25034 (Year Structure Built)	(B25034_007E + B25034_008E)/B25034_001E
poverty	S1701 (Poverty Status)	S1701_C03_001E/100

The three era shares are combined into a single housing‐age score that weights older construction more heavily:

$$A=100\;\left(0.619\;s_{40}+0.309\;s_{4059}+0.075\;s_{6079}\right)$$

The band weights (0.619, 0.309, 0.075) decline with construction age, weighting the oldest and highest‐lead era most. They are a hand‐chosen monotone set, close to a simple 8:4:1 ratio, not derived from any published formula and not fitted to blood lead; the motivation is only the established fact that lead‐based paint is far more prevalent in older housing. Because the housing‐age score is *z*‐scored before it is combined with poverty, the overall scale of the three weights drops out and only their ratios carry through. Their justification is the weight‐sensitivity analysis below, which shows the validation is insensitive to the exact split as long as older stock is weighted more heavily, so no result in this paper rests on these particular values. The housing‐age score and poverty are each standardized to a *z*‐score across the scored national universe, so the two share a common scale before they are weighted:

$$z\left(x\right)=\frac{x-\bar{x}}{s_{x}}$$

The standardized housing‐age score and standardized poverty are combined into a composite risk score, which is converted to a within‐nation percentile rank:

R=0.58z(A)+0.42z(poverty)

$$P=100\times \frac{\text{rank}\left(R\right)}{N}$$

Here *A* is the housing‐age score, *z*(*x*) is the standardization above, *R* is the composite risk score, rank(*R*) is the ascending rank of *R* across the *N* scored units, and P is the published 0‐to‐100 score. A unit at the 90th percentile carries higher predicted risk than 90% of scored units nationally.

The 0.58/0.42 composite split weights housing age above poverty, reflecting that housing age is the proximate source of the lead while poverty modifies exposure and remediation (CDC, n.d.‐f). Neither the band weights nor the composite split is fitted to the blood‐lead data. The Washington State Department of Health index weights housing age and poverty and bins the result to deciles; we keep the full 0‐to‐100 resolution (Washington State Department of Health, n.d.). To confirm the result does not depend on these choices, we re‐derived the index across a grid of weightings and recomputed every validation correlation, reported under Weight sensitivity below. Because the score is ordinal by construction, the validation uses rank correlation.

After dropping records with a missing or zero housing denominator and Census‐suppressed cells (described above), the method scored **3,222 counties** and **83,388 census tracts**. This is the subset of the full 2018–2022 ACS tract universe with a valid housing‐unit denominator. For comparison, the EPA hotspots analysis screened 73,086 tracts on the older ACS 2013–2017 geography, restricted to tracts in the 50 states containing at least one child under 6 years old (Zartarian et al., 2024). Our larger count reflects the newer 2020‐vintage tract geography and the inclusion of tracts regardless of child presence. The 83,388 scored tracts cover the 50 states and the District of Columbia. The 3,222 counties and county‐equivalents also include the District of Columbia and Puerto Rico's municipios, which is why Puerto Rico appears in the county outputs but not the tract outputs. The scored national surface is shown in Figure 1.

**Figure 1:**
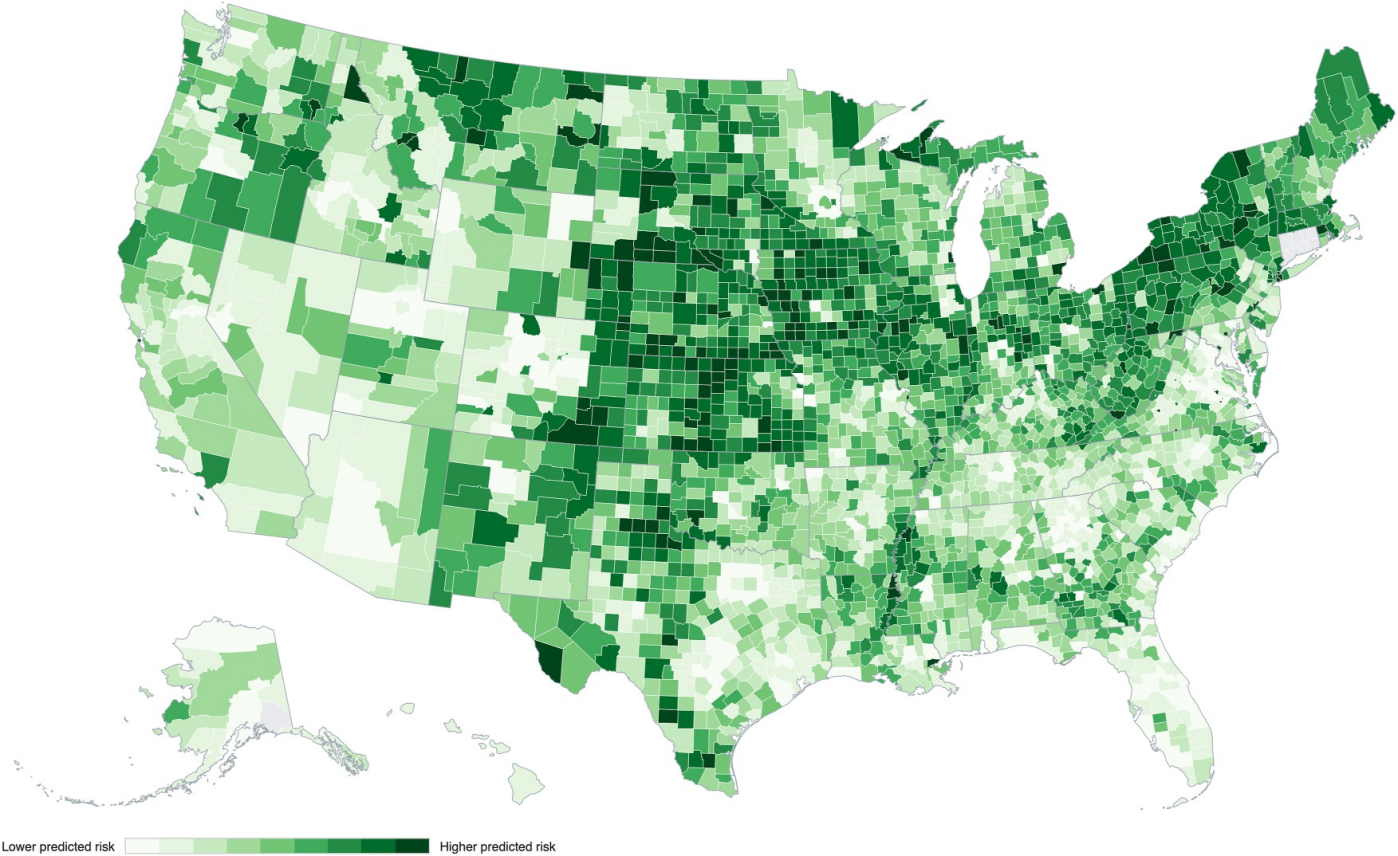
The national lead‐exposure risk map, shown at the county scale. Every U.S. county and census tract is scored from public Census housing‐age and poverty data and percentile‐ranked; counties are shaded from light (lower predicted risk) to dark green (higher predicted risk). Older‐housing regions of the Northeast, Midwest, Great Plains, and Appalachia rank highest, and the newer‐built West and Sun Belt rank lower. Projection is Albers USA, with Alaska and Hawaii repositioned. The full interactive version, including the census‐tract layer, is freely available online.

We did not impose a child‐presence filter on the score itself; the B01003 total population is carried alongside each scored unit for population‐weighting (U.S. Census Bureau, 2023e).

### 2.5 Scope and Interpretation

The output is a prediction of relative risk from housing age and poverty. It does not measure lead in any specific home, and it does not diagnose any child. It is a screening surface meant to direct confirmatory testing, consistent with EPA's own statement that “there is no known level of lead exposure to be without risk” and with the use of these indices as targeting tools rather than exposure measurements (CDC, n.d.‐f; Zartarian et al., 2024). The entire pipeline is reproducible from the four ACS tables and the public Census API with no restricted or licensed inputs.

### 2.6 Validation

A risk map is only worth deploying if it agrees with where children actually carry elevated blood lead. We tested ours in two stages. First we summarize the federal anchor: the EPA hotspots analysis, which validated housing‐and‐poverty indices against roughly 4.2 million measured childhood blood‐lead tests in two states. Then we test our published map against measured childhood blood lead in 11 states; eight of them form the main design, in three distinct tiers. Michigan and Ohio, at the census‐tract scale, reuse the source study's own surveillance, so they are a consistency check on its ground truth rather than an independent test. Wisconsin, also at the census‐tract scale and now statewide across 71 counties, is the independent test at tract resolution, on surveillance the source study never used. Five more states (New Jersey, Illinois, Missouri, Iowa, and New York) are independent tests at the coarser county scale. Three further independent states (Massachusetts, Minnesota, and Rhode Island, at the tract scale) are shown in Figure 2 with the headline states, and finer‐scale ZIP checks in Missouri and New York are scored the same way and detailed below. We report the tract and county scales separately and never pool them into a single figure. The two stages also use different statistics: the federal work reports Cohen's kappa for binary hotspot agreement, and we report the Spearman rank correlation between continuous predicted risk and continuous measured exposure. We keep the two separate and do not convert one into the other.

**Figure 2:**
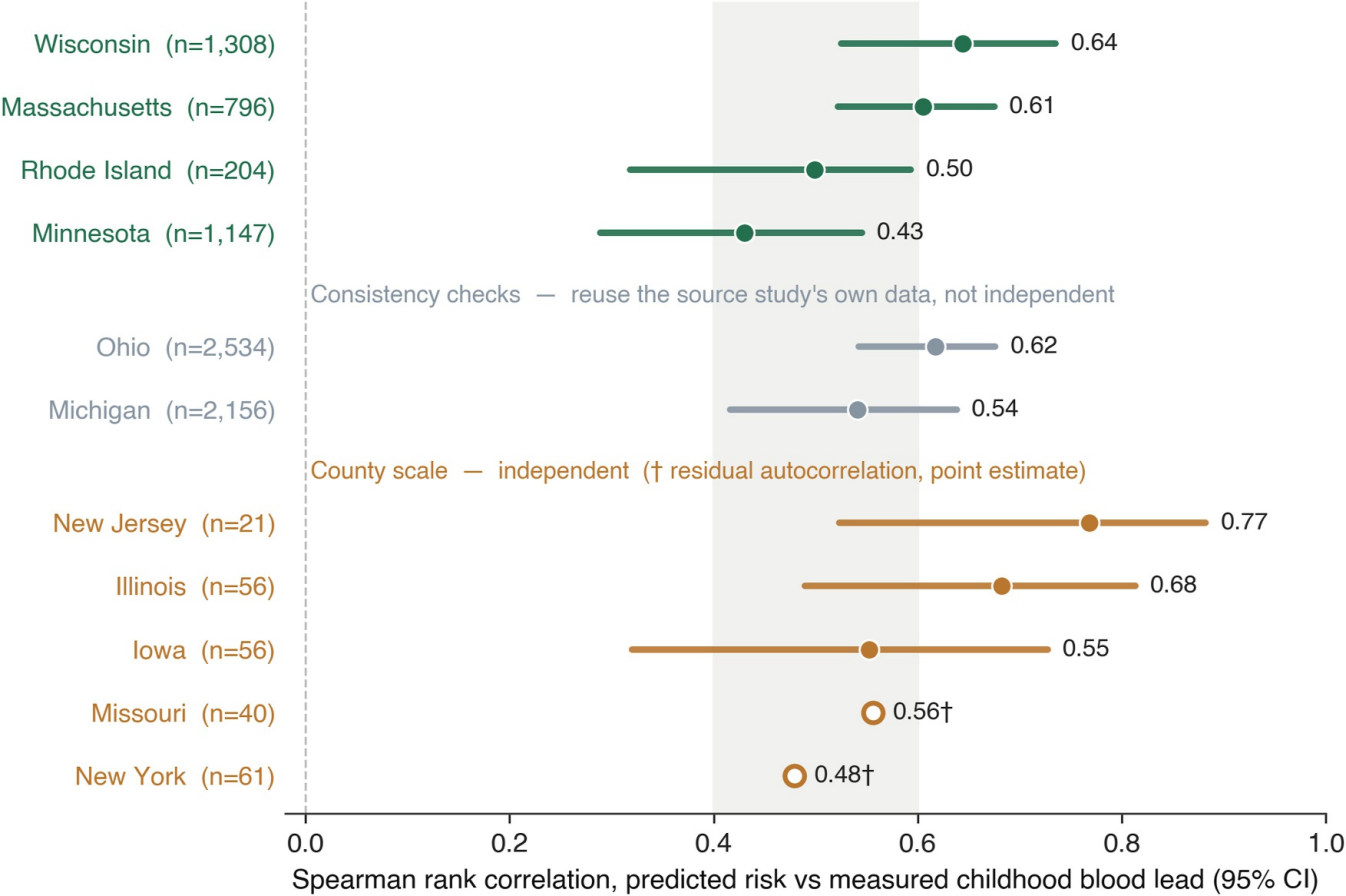
Spearman rank correlation between predicted neighborhood risk and measured childhood blood lead, by state. Green marks the four independent tract‐scale tests, on surveillance the source study never used: Wisconsin (statewide, 1,308 tracts across 71 counties), Massachusetts, Rhode Island, and Minnesota, each with a spatial block bootstrap 95 percent interval. Gray marks the two tract‐scale consistency checks, Michigan and Ohio, which reuse the source study's own surveillance and are not independent. Amber marks the five independent county‐scale states, with intervals from resampling counties; a dagger (†) marks Missouri and New York, whose residuals carry spatial autocorrelation, so their resampling intervals understate the uncertainty and they are reported as point estimates. The shaded band is the conventional moderate range of 0.4–0.6. County correlations are not directly comparable to tract correlations, because a coarser areal unit tends to raise them.

### 2.7 Is the Validation Circular?

The first objection to a housing‐based risk map is that it may be near‐tautological: the index is built mostly from the age of the housing stock (housing age carries the larger composite weight, 0.58, and within it the pre‐1940 band is weighted most heavily), and measured childhood blood lead is itself driven largely by old leaded paint, so predicted risk and measured exposure could appear to agree by construction. Three features of the design answer that objection. The central test is out of sample: the index weights are fixed in advance and never fit to blood lead, and the independent comparisons (Wisconsin statewide at the tract scale and five states at the county scale) use surveillance the index was never tuned to, where agreement is not guaranteed. The agreement also survives inside counties: with county means removed from the Wisconsin tracts the within‐county correlation is still 0.50, so the index separates neighborhoods within a county, not merely old industrial cities from new suburbs, which a tautology resting on coarse geography could not do. If the agreement were instead a built‐in identity the rank correlation would approach 1.0, yet even a flexible model given the same public inputs explains only about half the tract‐level variance (R‐squared 0.45 to 0.48 under county‐grouped cross‐validation, Residual analysis), so most of the variation is genuinely unexplained by housing age and poverty. And the weight‐sensitivity analysis shows the ranking is not an artifact of a tuned split, while the predictors lead the measured outcomes by roughly a decade, which attenuates a correlation rather than inflating it. The map does not claim to measure lead; it ranks where measured testing should go first, and the validation asks only whether that ranking matches where children actually test high.

### 2.8 The Federal Anchor: Zartarian et al. (2024)

The scientific foundation is Zartarian et al., “A U.S. Lead Exposure Hotspots Analysis,” published in *Environmental Science & Technology* in 2024. EPA's Office of Research and Development screened **73,086 census tracts** containing at least one child under six across all 50 states, scoring each tract on lead‐exposure indices built from housing age and sociodemographic data drawn from the American Community Survey.

The validation against measured childhood blood lead, rather than the prediction by itself, is what gives that paper its weight. EPA held the predicted hotspots against measured childhood blood‐lead surveillance in two states with unusually complete records:

- *Michigan*: approximately **1.9 million** blood‐lead results from children under six, covering **2006 to 2016** (Zartarian et al., 2024).
- *Ohio*: approximately **2.3 million** blood‐lead results from children under six, covering **2005 to 2018.**

Across those roughly 4.2 million measured tests, the predicted hotspots agreed moderately to substantially with where children actually carried elevated blood lead, at **Cohen's kappa of 0.49 to 0.63** (Zartarian et al., 2024). EPA read kappa on a fixed scale: below 0.4 low, 0.4 to 0.6 moderate, 0.6 to 0.8 substantial, above 0.8 near‐perfect. A companion tract‐scale study of Ohio by the same EPA group reports a comparable band, kappa **0.54 to 0.64** between observed elevated‐blood‐lead hotspots (Getis‐Ord Gi*) and the predictive indices it evaluated (Stanek et al., 2024).

Two further results matter for anyone building on it. First, a reduced **three‐variable model**, using only percent of homes built before 1940, percent built before 1950, and the percent of families with an income‐to‐poverty ratio above 2, performed comparably to the full five‐variable model, holding kappa at **0.51 to 0.63** across the Michigan and Ohio data sets (Zartarian et al., 2024). Housing age plus poverty account for most of the predictive power, which is what makes a transparent, reproducible national map possible from public data alone. Second, the authors anchor the entire effort in the established toxicology, stating plainly that “there is no known level of lead exposure to be without risk” (Zartarian et al., 2024). A screening map does not need to find a safe threshold, because none exists; it only has to rank where exposure concentrates.

### 2.9 Our Method, in Brief

Our national map combines housing age and poverty, the two determinants the source study found carry most of the predictive signal. Housing age comes from ACS table **B25034 (Year Structure Built)** as three construction‐era shares weighted toward older stock, because older homes carry the highest lead‐paint burden (U.S. Census Bureau, 2023f). Poverty comes from ACS table **S1701 (Poverty Status in the Past 12 Months)** (U.S. Census Bureau, 2023d). We standardize the housing‐age score and poverty, weight them 0.58 to 0.42, and sum. Tracts are then percentile‐ranked within the national pool of 83,388 tracts; for the county‐scale validation the same index is computed on county totals and ranked within the national pool of 3,222 counties. This follows the Washington State Department of Health housing‐age‐plus‐poverty lineage rather than introducing a new index. The full equations are in Methods.

### 2.10 Validation at the Census‐Tract Scale

For each state we joined predicted tract‐level risk to measured childhood blood lead at the tract level, then computed the **Spearman rank correlation**. Because the published national percentile is a monotone, rank‐preserving transform of the composite score, and the Spearman correlation depends only on within‐state ranks, the within‐state rank correlation of the percentile equals that of the underlying score; ranking tracts nationally to publish the map therefore introduces no ranking artifact in any within‐state validation result. Neighboring tracts are spatially autocorrelated, so an ordinary bootstrap that resamples tracts one at a time treats correlated observations as independent and returns an interval that is too narrow. We instead use a spatial block bootstrap: tracts are partitioned into compact geographic blocks by a quantile grid on their centroids, and whole blocks are resampled with replacement, 3,000 times. We also report Moran's I of the regression residual (observed on predicted) under queen contiguity, with a permutation *p*‐value, as a direct measure of the leftover spatial structure the block bootstrap is correcting for.

What counts as independent differs by state. For Michigan and Ohio the measured values come from the same surveillance the source study used, so these two are a reproduction of the source result on its own ground truth, a consistency check rather than an independent test. The Michigan and Ohio measured values are the census‐tract childhood blood‐lead exceedance rate, defined in both source analyses as the number of children tested with an elevated result divided by the number of children tested in the tract, times 100, a tested‐children denominator rather than a population denominator, for children under six, from the state surveillance the source study used, documented for Michigan by Xue et al. (2022) and for Ohio by Stanek et al. (2024). The elevated threshold in those sources is the older 5 μg/dL reference value (the Ohio companion also reports 3.5 and 10 μg/dL); because this validation uses rank correlation, the threshold choice does not materially affect the result, since a tract's rank by exceedance rate is nearly unchanged across thresholds. These two outcome files are static extracts from those published analyses and are the one part of the pipeline without a live generator; every other measured source is pulled by a script in the repository. Wisconsin is an independent test, statewide across 71 counties: its measured tract data come from the Wisconsin DHS open ArcGIS service, which we pulled live with no records request.

**Table:** 

State	Measured source	Tracts (n)	Spearman ρ (95% CI)
Michigan	source‐study surveillance, 2006–2016 (consistency check)	2,156	**0.54** (0.42–0.64)
Ohio	source‐study surveillance, 2005–2018 (consistency check)	2,534	**0.62** (0.54–0.68)
Wisconsin (statewide, 71 counties)	WI DHS open ArcGIS, children under 6	1,308	**0.64** (0.53–0.74)

All three correlations are positive, with spatial block bootstrap intervals well clear of zero. The residual carries strong positive spatial autocorrelation in every case (Moran's I 0.49 in Michigan, 0.53 in Ohio, 0.56 in Wisconsin, all permutation *p* ≈ 0.001), which is exactly why the ordinary tract‐level bootstrap intervals (0.51–0.57, 0.59 to 0.64, and 0.61 to 0.68) are too narrow and the wider block intervals above are the ones to trust. A county‐block bootstrap, resampling whole counties rather than grid cells, gives intervals consistent with the grid blocks in all three states (Michigan 0.39 to 0.64, Ohio 0.51 to 0.70, Wisconsin 0.45 to 0.75). Because the Wisconsin data are now statewide across 71 counties, the county‐block resample corroborates the Wisconsin interval as well, which an earlier Milwaukee‐only version could not do. The Wisconsin data come from the state DHS open ArcGIS service, which publishes children under six tested, children testing positive, and percent poisoned by tract; a tract is suppressed when fewer than five children are poisoned and fewer than 100 are tested, and we drop those suppressed tracts rather than treat the negative suppression sentinel as a rate (Wisconsin Department of Health Services, n.d.).

A rank correlation of 0.54–0.64 means the map's ordering and the measured ordering move together moderately, but much of the variation is unexplained: those correlations share roughly a third of the rank variance with measured exposure, leaving the rest to factors outside housing age and poverty. A gradient‐boosted model fit to the same public inputs, cross‐validated within Michigan and Ohio, reaches an R‐squared of about 0.45–0.48 (see Residual analysis); that is an in‐distribution upper bound for this feature set in those two states, rather than a national bound or the fixed‐weight index's own fit. This is a screening signal and not a diagnosis, and it says nothing about any individual child's blood lead.

Every validation set is defined by which tracts have non‐suppressed measured blood lead, so each comparison is conditional on measured coverage. After dropping suppressed tracts we retain 2,156 of 2,896 measured tracts in Michigan (a 25.6% drop), 2,534 of 3,151 in Ohio (19.6%), and 1,308 of 1,524 in Wisconsin (14.2%). The direction matters: in Michigan and Ohio the dropped tracts sit at systematically lower predicted risk than the kept ones (median percentile 0.47 vs. 0.62 in Michigan, 0.42 vs. 0.70 in Ohio, Mann‐Whitney *p* < 0.001), so those two correlations describe a higher‐risk, range‐restricted subset of the published surface. Wisconsin, the state that carries the independent tract claim, shows no such difference between dropped and kept tracts (*p* = 0.58), and the testing‐coverage analysis below shows the correlation strengthens in better‐tested tracts, the opposite of what selection‐inflated agreement would produce.

### 2.11 Validation at the County Scale: Five More States

We also ran a coarser but broader test at the **county** level. Here the predictor is a county‐resolution version of the same index, computed from county housing‐age and poverty (the identical three‐band housing‐age score and 0.58/0.42 composite) and percentile‐ranked within the national pool of 3,222 counties, not an average of tract scores. We joined it to measured childhood blood lead from the CDC Environmental Public Health Tracking Network 2022 series (children under six, at or above the 3.5 μg/dL reference value). All five use surveillance the source study did not, so they are independent tests, and all five share the same 3.5 μg/dL threshold and 2022 vintage. The CDC series reports only counties with sufficient testing, so each is a subset of the state's counties (New Jersey 21 of 21, New York 61 of 62, Illinois 56 of 102, Iowa 56 of 99, Missouri 40 of 115); the correlation is conditional on CDC‐reporting counties, which skew more populous and better tested.

**Table:** 

State	Counties (n)	Spearman ρ (95% CI)
New Jersey	21	**0.77** (0.52–0.88)
Illinois	56	**0.68** (0.49–0.81)
Missouri	40	**0.56** (0.29–0.73)
Iowa	56	**0.55** (0.32–0.73)
New York	61	**0.48** (0.22–0.69)

These intervals come from resampling counties, which are themselves the spatial unit, so unlike the tract intervals they are not separately corrected for spatial autocorrelation. The residual carries little county‐to‐county autocorrelation in Illinois, Iowa, and New Jersey (Moran's I near zero, not significant), but it is substantial in Missouri and New York (Moran's I 0.59 and 0.48, permutation *p* ≈ 0.001). For those two states the county resample understates the uncertainty, so we report Missouri and New York as point estimates and state the headline county range over the three states whose residuals show no significant autocorrelation: New Jersey, Illinois, and Iowa. County correlations should also not be read on the same scale as the tract ones: a coarser areal unit averages over within‐county variation and tends to raise the correlation, an instance of the modifiable areal unit problem. The small county counts give wide intervals, widest for New Jersey at *n* = 21. New York is the weakest of the five. The CDC Tracking Network county series we use for New York covers all five New York City boroughs; the separate ZIP‐level Socrata series described below is the one that excludes New York City.

We apply one inclusion rule uniformly: a county series enters the headline only if it uses the current 3.5 μg/dL reference value and its correlation is distinguishable from zero, meaning a 95% confidence interval that excludes zero. Every county series above passes. The one county series that does not is Massachusetts, on the older 5 μg/dL threshold and only 13 counties, where *ρ* = 0.42 with an interval from −0.20 to 0.86 that includes zero; we leave it out of the county figure. Its far richer tract‐scale surveillance is a separate matter and gives a strong independent result, reported in the additional‐states table below.

Across the independent tests, Wisconsin statewide at the tract scale and the five county states, predicted risk tracks measured childhood blood lead at a moderate level (Figure 2), on surveillance systems the source study never used. We do not compare these rank correlations against the source study's kappa, and we do not average the tract and county correlations into one figure, because neither pair measures the same thing.

### 2.12 Additional Independent States

Massachusetts, Minnesota, and Rhode Island, the three additional independent tract states shown in Figure 2, come from open services on surveillance the source study never used. We pulled each live, scored it with the same housing‐age and poverty index, and gave it the same spatial treatment the headline states get: a spatial‐block bootstrap interval and the residual Moran's I. They differ from Michigan, Ohio, and Wisconsin in vintage and elevated‐blood‐lead threshold, noted in the table below, but every interval clears zero and every residual carries the same positive spatial autocorrelation. The table below also includes a finer‐scale ZIP check in Missouri.

**Table:** 

State	Scale	Measured source (vintage)	n	Spearman ρ (95% CI)	Residual Moran's I
Massachusetts	tract	MA DPH via Harvard CGA, rate per 1,000, 2012‐2017	796	0.61 (0.52–0.68)	0.39
Missouri	ZIP	MO DHSS (official), elevated per children tested, 2023	194	0.60 (0.48–0.69)	0.57
Rhode Island	tract	RI DOH (official), elevated rate by tract	204	0.50 (0.32–0.59)	0.16
Minnesota	tract	MN DOH (official), percent elevated, 2016‐2020	1,147	0.43 (0.29–0.55)	0.29

Intervals are the spatial‐block bootstrap (county blocks for the tract states, a quantile grid for Missouri's ZIP geography, matching the headline method); all four clear zero. Massachusetts is the strongest, a second large independent tract state at 0.61, and Minnesota spans 86 counties, the broadest single‐state coverage in the paper. Minnesota, Rhode Island, and Missouri come from official state health‐department open services; Massachusetts is a Harvard Center for Geographic Analysis data set built on Massachusetts DPH childhood‐lead surveillance, the one research‐hosted source in the set. It is also the one source expressed as a rate per 1,000 children rather than a percent of children tested, so part of its agreement could track where testing is densest; we keep it because it is the second‐largest independent tract set and its interval clears zero, but its denominator differs from the others and should be read with that in mind. Missouri uses the New York method, the housing‐and‐poverty index computed on ACS ZIP code tabulation areas. The pulls and scores are reproduced by additional_states.py in the repository.

### 2.13 Screening Performance

Rank correlation measures whether the ordering is right, but a screening layer is also judged on yield: if a program flags the highest‐risk tracts first, how much of the summed elevated‐rate does it reach. We computed this from the same validation joins, ranking tracts by predicted risk and walking down (screening_performance.py).

The top decile of predicted risk carries 3.2 to 3.8 times the statewide mean elevated rate (Michigan 3.2, Ohio 3.3, Wisconsin 3.8). Flagging the top 20% of tracts reaches about half of the summed elevated‐blood‐lead rate (Michigan 50%, Ohio 53%, Wisconsin 55%), against the 20% an unranked list would reach, a 2.5–2.7‐fold concentration. Treated as a classifier for the worst‐affected tracts, the top quintile of measured elevated rate, the map separates them from the rest with an area under the curve of 0.86 in Michigan and 0.89 in Ohio and Wisconsin. At the county scale the same yield is present but coarser and weaker, strongest in New Jersey (AUC 0.91) and Iowa (0.83) and weakest in Missouri (0.62). These capture figures weight tracts equally; a child‐count‐weighted capture would need per‐tract tested or positive counts, which the public joins do not carry and a registry holds, so we leave that finer version to the address‐level confirmation we invite in the Discussion.

The tract‐scale signal is not one city. The single largest county holds 14 to 22% of each state's validation tracts, and dropping it entirely leaves the correlation positive and material: Michigan falls from 0.54 to 0.45 without Wayne, Ohio from 0.62 to 0.56 without Cuyahoga, Wisconsin from 0.64 to 0.52 without Milwaukee. Decomposing Wisconsin, the between‐county correlation across the 71 county means is 0.55 and the within‐county correlation after removing those means is 0.50, so the index discriminates both across counties and within them, not only along the Milwaukee‐versus‐rest gradient; Michigan and Ohio show the same pattern (between‐county 0.41 and 0.55, within‐county 0.47 and 0.55). A meaningful share of the raw tract correlation is still the between‐county gradient, which is expected for an ecological index and is reported here rather than left implicit.

### 2.14 Comparison to Prior Public Risk Layers

The national map is not a new method, so the question is how it compares to the public risk layers already in use. Those layers are more alike than their separate names suggest: the Washington State Department of Health Lead Exposure Risk Index, the Vox map (Frostenson & Kliff, 2016), the City Health Dashboard Lead Exposure Risk Index, and PolicyMap's lead index are all built from the same two inputs, housing age and poverty, and differ mainly in the exact weighting, in binning to deciles, and in ACS vintage. The EPA reduced hotspots model uses three inputs: the pre‐1940 and pre‐1950 housing shares and the share of residents with an income‐to‐poverty ratio at or above two. We reconstructed these shared forms on the current ACS 2018–2022 vintage and correlated each, per state, against the same measured childhood blood lead, so that only the predictor changes (benchmark_comparison.py).

**Table:** 

Predictor (same tracts, same outcome)	Michigan	Ohio	Wisconsin
This index (housing age top‐loaded toward pre‐1940, plus poverty)	0.54	0.62	0.64
Shared housing‐age + poverty form (WA‐DOH/Vox/City Health Dashboard/PolicyMap), equal weight	0.51	0.59	0.62
The same shared form binned to deciles, as those layers publish it	0.50	0.58	0.62
EPA reduced‐model inputs (pre‐1940, pre‐1950, income‐to‐poverty ratio ≥2)	0.57	0.63	0.65

Two things follow, and neither is a claim of a better algorithm. First, weighting older housing more heavily than the legacy equal‐weight maps adds a small, consistent gain, about 0.02–0.03 in rank correlation across the three states, and binning to deciles, which those maps do, costs a little resolution. Second, the EPA reduced‐model input set, which substitutes an income‐to‐poverty ratio for the below‐poverty share, edges this index by 0.01–0.03; this is consistent with the residual analysis, where richer socioeconomic variables add a little. We keep the simpler below‐poverty share for transparency and because the difference sits inside the validation's own uncertainty. The point of the table is not that this map wins. It is that a transparent, fixed‐weight index computed for every tract from two public variables performs on par with the federal reduced model and above the legacy public layers, on ground truth none of them was tuned to. We could not run EPA's actual trained random forest or PolicyMap's subscription‐only layer; for those two, because all of these public layers share the same two inputs, the reconstruction stands in for their functional form rather than copying any one vendor's exact output.

The City Health Dashboard layer is the exception: it publishes actual values, so we pulled its Lead Exposure Risk Index directly through its API and compared the real layer instead of a reconstruction. City Health Dashboard publishes only for tracts inside roughly the thousand largest cities, an urban subset, and because the predictor‐outcome relationship is stronger in dense urban tracts, comparing its urban values against our statewide index would flatter it. Recomputing every predictor on exactly the tracts it covers (900 in Michigan, 878 in Ohio, 527 in Wisconsin), our index matches or slightly exceeds the published City Health Dashboard layer: 0.73 versus 0.73 in Michigan, 0.76 versus 0.74 in Ohio, and 0.78 versus 0.77 in Wisconsin, with the EPA reduced‐model inputs again a touch ahead of both. Every predictor scores near 0.7 to 0.78 on this urban subset, against 0.5 to 0.64 statewide, which is exactly why an urban‐only layer cannot be set head to head against a statewide one without first restricting to shared tracts. Stated on that shared coverage, the result is the contribution precisely: the national index performs at least as well as the actual deployed City Health Dashboard layer where the two overlap, and carries the same ranking to the rural tracts that layer leaves out.

### 2.15 Sensitivity to the Weighting

The composite split (0.58 housing, 0.42 poverty) and the housing‐band weights (0.619, 0.309, 0.075) are set a priori, not fitted, so a fair question is whether the result depends on them. We re‐derived the index from the raw national ACS distribution across nine weighting schemes and recomputed each tract‐scale correlation. The published weights reproduce the canonical values exactly (Michigan 0.54, Ohio 0.62, Wisconsin 0.64). Moving the composite split anywhere from 0.42/0.58 to 0.70/0.30 changes each correlation by less than 0.03. The housing‐band weighting matters more, and in the expected direction: weighting older stock more heavily, as the published weights and a pre‐1940‐only weighting both do, outperforms weighting the three construction eras equally, which drops the correlation to 0.45 in Michigan, 0.53 in Ohio, and 0.57 in Wisconsin. Dropping either housing age or poverty entirely is worse than keeping both. The ranking is driven by the prior decision to weight older housing more heavily and to include poverty, a direction grounded in the lead‐paint literature, not by the precise split, which is a hand‐chosen monotone set the sensitivity grid shows does not drive the result.

### 2.16 Sensitivity to Tract‐Boundary Vintage

The index is built on 2020‐vintage census tracts (ACS 2018–2022), while the Michigan and Ohio measured data predate that geography, collected from 2006 to 2016 and 2005 to 2018 on 2010 tracts. A direct 11‐digit GEOID join can therefore mismatch tracts that split or merged between the two vintages. Using the Census 2010‐to‐2020 tract relationship file, 96.9% of the Michigan validation tracts and 97.5% of the Ohio tracts are unchanged one‐to‐one between vintages. Restricting each correlation to those unchanged tracts leaves it essentially identical (Michigan 0.54 on both the full and the unchanged set; Ohio 0.62 vs. 0.61), so the cross‐vintage join does not drive the result. The predictors are also more recent than these outcomes by roughly a decade, which attenuates a correlation rather than inflating it, so the tract‐scale figures are if anything conservative.

### 2.17 Sensitivity to Testing Coverage

Measured childhood blood lead comes only from children who are tested, and testing is targeted rather than universal, so a fair concern is that these correlations track where testing happens rather than where lead is. Two things bound that concern. First, every measured value we use is a rate, the percent of tested children who are elevated rather than a count of cases, so it is not mechanically inflated by testing more children. Second, where the data also publish the number of children tested, we condition on it.

At the census‐tract scale, the Wisconsin service reports children tested per tract. Statewide, predicted risk is modestly related to testing volume (Spearman 0.36). The assumption‐free check is to restrict to well‐tested tracts, where the rate is measured most precisely: there the correlation is if anything stronger (0.66 at 100 or more children tested, 0.77 at 200 or more), the opposite of a testing‐volume artifact. A rank‐based partial correlation controlling for testing volume agrees (0.59 vs. the raw 0.64). We use a nonparametric partial: rank‐transform predicted risk, the elevated rate, and testing volume, residualize the ranks of predicted risk and outcome on the rank of testing volume, and correlate the residuals. A partial correlation is not in general bounded by its zero‐order correlation; when the control variable is a suppressor it can sit slightly above the raw value, as New Jersey shows below.

At the county scale, we reconstructed the number of children tested per county from the CDC series (the elevated count divided by the elevated rate). In four of the five states, predicted risk is negatively correlated with testing volume (Spearman −0.16 to −0.57; New Jersey is the exception at 0.34), which means the highest‐risk counties test the fewest children. That is the surveillance gap the map is built for: measurement is thinnest exactly where predicted risk is highest. Controlling for testing volume with a rank‐based partial, the predicted‐risk‐to‐elevated‐rate correlation stays positive in every state (0.36 in Iowa, 0.48 in Missouri, 0.56 in Illinois, 0.55 in New York, 0.79 in New Jersey). New Jersey's partial sits just above its raw correlation (0.79 vs. 0.77), a genuine suppression effect rather than an artifact: testing volume is moderately correlated with predicted risk there (rho 0.34) but essentially uncorrelated with the elevated rate (rho 0.07), so removing the testing‐correlated part of predicted risk slightly sharpens the association. Every other state's partial sits at or below its raw correlation, as expected when the highest‐risk counties are also the least‐tested. The reconstructed tested denominator is undefined where the elevated count or rate is zero or suppressed, which drops Illinois to 55 and New York to 58 counties for this coverage check, against the full headline sets of 56 and 61. The attenuation in some states reflects that lower‐testing counties are also higher‐risk, a shared rural and disinvestment gradient, rather than an artifact in the rate itself.

A validation fully independent of who gets tested would require population‐representative testing, such as NHANES, or a complete state registry rather than the public aggregate extracts used here. We invite that confirmation (see Discussion).

### 2.18 A Finer‐Scale Independent Check: New York ZIP Codes

New York publishes childhood blood‐lead counts by ZIP code, excluding New York City, which lets us test the same index at a finer scale than the county series, on data the source study never used. We pooled counts from 2018 forward by ZIP and correlated the measured elevated rate against the housing‐age and poverty index, z‐scored within the matched ZIP set (New York State Department of Health, n.d.).

Elevated blood lead is rare at this scale. With a 50‐test floor, 93% of the ZIPs record zero elevated children, so the measured rate is mostly ties at zero and a rank correlation is held down by sampling noise rather than by the model. As the minimum number of tested children rises and the rate is measured more precisely, the correlation climbs from 0.36 at 50 tests to 0.69 at 1,000, into the tract‐scale range among the best‐measured ZIPs.

**Table:** 

Minimum children tested	ZIPs (*n*)	Share with zero elevated	Spearman ρ
50	1,119	93%	0.36
100	895	91%	0.40
200	646	88%	0.47
500	386	80%	0.59
1,000	232	71%	0.69

This is a corroborating check and not a headline number. It is one state, ZIP codes are coarser and messier than census tracts, and the series excludes New York City, so its spread of housing age and poverty is narrower than the state as a whole. It shows the same ordering holding at a finer grain in a second independent surveillance system. One caveat applies to every ZIP check, here and in the additional‐states table: the ZIP predictor is z‐scored within the matched ZIP set rather than read from the national percentile surface, so it tests the same index form rather than the exact published ranking, and local standardization shifts the balance between housing age and poverty by their within‐set spread.

### 2.19 The Data‐Access Pipeline, and Why Prediction Is Necessary

Measured childhood blood‐lead is held by states, and access varies sharply from one to the next. That is the practical reason a prediction map is useful: it brings the same neighborhood‐level warning to every state, including the many that publish no usable blood data at all.

Where measured data exists in machine‐readable form, we ingest it automatically:

- *New York (ZIP‐level*, *Socrata)*. New York publishes childhood blood‐lead testing and elevated‐incidence counts by ZIP code (excluding New York City) on health.data.ny.gov, served through the Socrata Open Data API (SODA) (New York State Department of Health, n.d.; Washington State Department of Health, n.d.). We query it programmatically.
- *County‐level*, *roughly 45 states (CDC Tracking Network API)*. The CDC Environmental Public Health Tracking Network exposes childhood blood‐lead surveillance through a machine‐readable API, with the **≥3.5 μg/dL** classification adopted for 2022‐forward data after CDC lowered the blood‐lead reference value from 5 to 3.5 μg/dL in October 2021 (CDC, 2021b). Children are counted once per year at their highest result (CDC, n.d.‐b). This is the broad county‐level backbone.
- *Wisconsin (tract‐level*, *statewide*, *ArcGIS)*. Pulled live from the WI DHS statewide service, as described above (Wisconsin Department of Health Services, n.d.).

Then there are the holdouts. States including *New Hampshire*, *Colorado*, *and Connecticut* lock their childhood blood‐lead behind Tableau dashboards or PDF reports with no API, so obtaining tract‐ or ZIP‐level measured values requires a public‐records (FOIA) request and manual extraction. These are exactly the places where a family has no public way to learn that their neighborhood's housing stock and poverty profile put their child at elevated risk. The prediction map closes that gap. It runs without waiting for a state to publish blood tests, because it needs none: only the public Census housing‐and‐poverty data, which exists for every tract in the country. The validation above is what justifies trusting that prediction where no measured data is available to check it.

### 2.20 Residual Analysis: How Much Further Public Data Can Go

A screening map should be honest about its own error. Two questions follow from the validation: why does predicted risk track measured blood lead at a rank correlation near 0.6 rather than 1.0, and would more public variables push it higher. We tested both in the two states behind the federal anchor.

### 2.21 What We Did

We assembled every census tract in Michigan and Ohio carrying both a published measured childhood blood‐lead value and complete Census inputs: 4,690 tracts, population‐weighted. We compared two models that predict measured blood lead from public variables, each scored by five‐fold cross‐validation within this two‐state set, so every reported figure is from held‐out tracts the model did not train on. The figures below therefore describe held‐out tracts within Michigan and Ohio, the two consistency‐check states whose surveillance the source study already used, not generalization to new states. Both models are gradient‐boosted regressions, and the analysis is reproducible from the public Census API pulls and the published state blood‐lead joins. The published risk index itself has fixed weights and is never fit to blood lead; the cross‐validated models here are auxiliary, used only to ask how much additional public data could improve the ranking. The baseline uses three inputs that parallel the EPA reduced model: the share of homes built before 1940, the share built before 1950, and poverty. We use the tract below‐poverty share for the socioeconomic term; the EPA reduced model used the share of families with an income‐to‐poverty ratio above 2, the same axis with the opposite sign. The expanded model adds seven more public variables: median home value, median household income, renter share, vacancy rate, the 1950‐to‐1979 housing share, percent Black, and percent Hispanic.

### 2.22 Housing Age Is Most of the Signal, and More Variables Barely Move the Ranking

Adding seven variables raised the rank correlation by 0.01 and the variance explained by 0.03 under county‐grouped cross‐validation. The R‐squared values above are scored by five‐fold cross‐validation grouped by county (GroupKFold on county FIPS); under random folds the same models reach 0.482 and 0.550, but residual Moran's I of 0.49–0.56 makes random folds optimistic, because neighboring train and test tracts leak spatial structure, and most of the apparent 0.07 gain is that leakage rather than transferable signal. The ranking is already close to the ceiling that public data can reach. A single input, the share of homes built before 1940, carries roughly 68% of the model's predictive weight (Figure 3a). This reproduces the EPA's own finding that a reduced three‐variable model performed comparably to its full model (Zartarian et al., 2024): housing age and poverty carry most of the signal, and further socioeconomic variables are largely redundant with them.

**Table:** 

Model	Inputs	Cross‐validated Spearman ρ	Variance explained (R^2^)
Baseline	3 variables (housing age + below‐poverty share)	0.603	0.448
Expanded	10 variables (adds race, value, income, tenure, vacancy)	0.617	0.478

**Figure 3:**
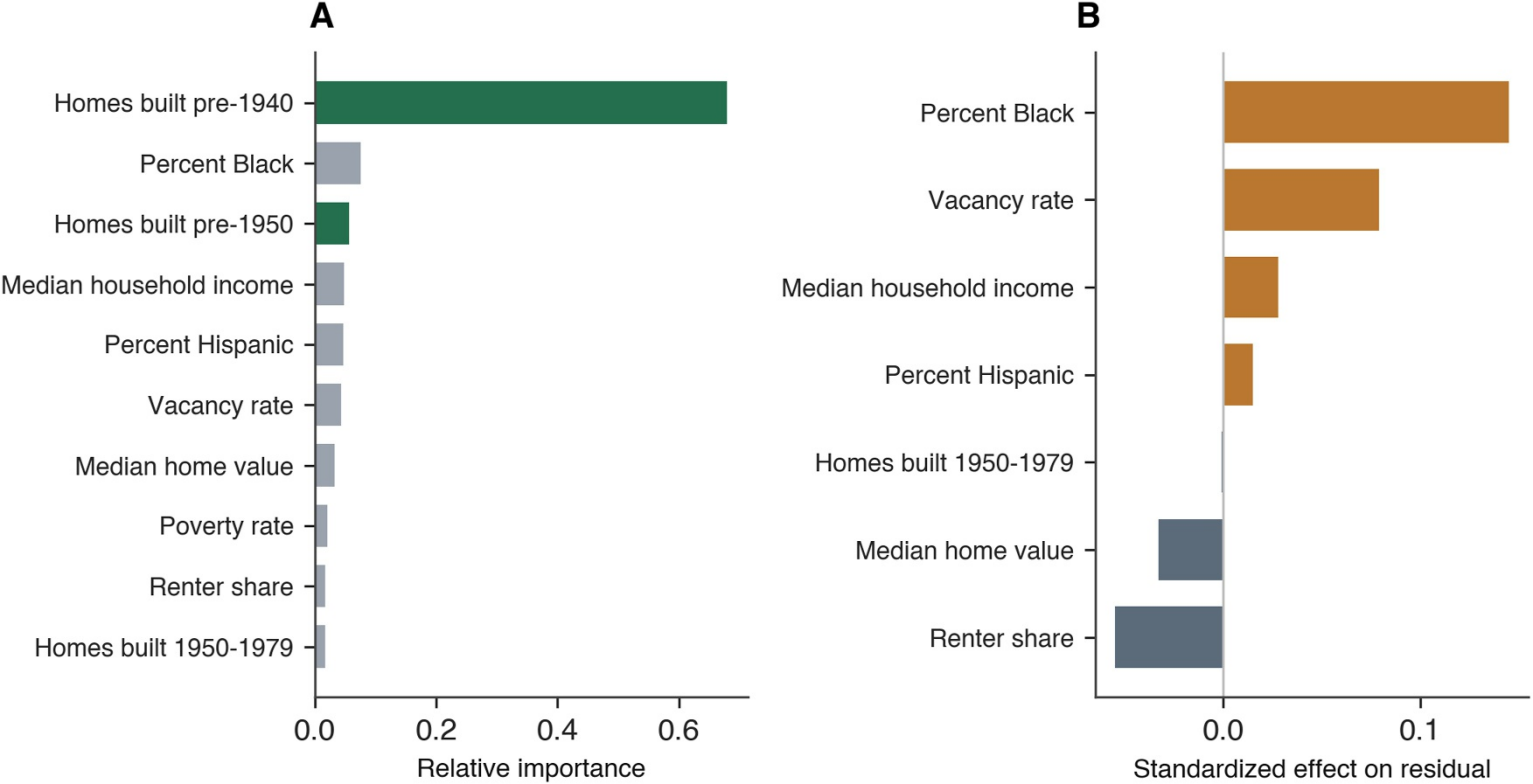
Residual analysis on 4,690 Michigan and Ohio tracts. (a) Relative importance of each public variable in a cross‐validated model of measured blood lead: the share of homes built before 1940 carries most of the signal. (b) Standardized association of each added variable with the residual the housing‐and‐poverty baseline leaves behind: percent Black population is the strongest single predictor of what the baseline misses in a ridge fit (standardized coefficient 0.145 vs. 0.079 for vacancy), though the marginal residual correlation is modest (*r* = 0.094); shown as a descriptive diagnostic on two states, not a causal claim.

### 2.23 Where the Map Under‐Predicts: A Residual Tracking Race and Disinvestment

We took what the housing‐and‐poverty baseline leaves unexplained, its out‐of‐sample residual, and asked which variables predict it (Figure 3b). Percent Black population is the strongest single predictor of the residual in a ridge fit (standardized coefficient 0.145, against 0.079 for vacancy and less for income), though its marginal correlation with the residual is modest (*r* = 0.094), because these neighborhood‐disadvantage predictors are collinear and the ridge fit spreads shared signal among them. Read that way, the map tends to under‐predict risk in tracts with larger Black populations and more vacant housing, relative to what housing age and poverty alone imply. This is a descriptive residual diagnostic on two states, not evidence of a causal pathway. It is consistent with the documented racial ecology of lead exposure, in which the legacy of redlining and disinvestment is thought to have concentrated deteriorating lead hazards in Black neighborhoods beyond what income or housing vintage captures (Sampson & Winter, 2016); we offer that as a hypothesis the pattern fits rather than a mechanism this analysis establishes.

We state the ethical constraint plainly. That race improves the statistical fit does not mean race should be an input to a deployed targeting tool. Allocating screening by race would risk encoding the disparity it measures. We report the result for the opposite reason: it is a descriptive diagnostic of where a housing‐and‐poverty map runs conservative, a signal that the map is more likely to under‐rank than over‐rank risk in the highest‐risk Black neighborhoods, though the residual association is modest (*r* = 0.094) and not a causal claim.

### 2.24 Why the Ceiling Exists, and What Is Beyond It

Three limits hold any ACS‐based map near a rank correlation of 0.6. Only one is fixable with more data.

First, the model is ecological. Every value is a property of a neighborhood rather than a home. A high‐risk tract contains remediated, lead‐free houses; a low‐risk tract contains pre‐1940 houses with failing paint. No neighborhood variable resolves a hazard that varies house to house. The constraint is the unit of analysis itself, the neighborhood, and not the variable list.

Second, the measured truth is itself noisy. A tract's blood‐lead rate rests on the children who happened to be tested, which in a small tract is few. Some of the unexplained variance is measurement error in the target, not error in the model.

Third, the remaining exposure pathways are absent from the census entirely. Lead service lines, soil lead, imported consumer goods such as glazed ceramics and certain spices, occupational take‐home lead, and the actual condition of paint are real drivers that no ACS table records.

The third limit points to two ways forward. The first is to combine external public data the census does not carry: the lead service‐line inventories that water systems were required to publish under the EPA Lead and Copper Rule Revisions in October 2024 (EPA, n.d.); parcel‐level assessor and sale records that proxy for maintenance and renovation; proximity to airports still burning leaded aviation fuel, now the largest source of airborne lead, which the EPA has formally found endangers public health (EPA, 2023); and soil‐lead surveys. Each is public, but fragmented and laborious to assemble, which is exactly why existing maps omit it. The second is physical confirmation in the home. A neighborhood model that has reached its data ceiling cannot tell a family whether their home holds a hazard; a direct test of the home, by a certified risk assessment, an XRF reading, dust‐wipe or paint‐chip lab analysis, or the child's blood‐lead test, can. Confirmation in the home is the step a map cannot replace.

### 2.25 Ethics and Human Subjects

This study used only aggregate, publicly available, de‐identified data at the census‐tract and county level. It involved no individual human subjects, no identifiable private information, and no intervention, and so did not require institutional review board approval. The analysis operates at the population (neighborhood) level and cannot determine exposure for any individual child or address.

An AI assistant (Anthropic's Claude) was used as a tool in this work: for retrieving and processing public data, for statistical and mapping code, for a first‐pass literature search, and for drafting and copy‐editing text. The author directed the work, wrote and audited the analysis pipeline, and re‐ran it to confirm every reported figure. Each cited reference was independently verified against its PubMed or CrossRef record before inclusion. The author takes full responsibility for the entire content. No AI system is an author, in keeping with ICMJE and publisher policy, because an AI cannot be accountable for the work.

## 3 Discussion: Potential Applications

### 3.1 Primary Prevention Means Starting With the House

Childhood lead policy distinguishes primary prevention, which keeps a child from ever contacting lead, from secondary prevention, which identifies a child who has already been exposed in order to limit further harm. In 2012 the CDC Advisory Committee on Childhood Lead Poisoning Prevention reviewed the evidence that no blood‐lead level in children is without effect and issued a renewed call to move the field's emphasis toward primary prevention (Advisory Committee on Childhood Lead Poisoning Prevention, 2012). Blood‐lead surveillance, the measured truth this map is validated against, is secondary by construction. An elevated result is measured in a child who has already absorbed the lead, and the exposure it records is not reversible (CDC, 2021a). A surveillance system that waits for that result documents harm after it has occurred rather than preventing it.

A neighborhood whose risk is predictable in advance does not need a child to serve as its first test. The determinants used here, the age of the housing stock and concentrated poverty, are published for every census tract in the country and are knowable before any child is screened. Where they flag elevated risk, the next step can be a test of the environment, a certified home risk assessment, an XRF reading, or a dust‐wipe or paint‐chip analysis, rather than a test of the child. That reordering, from the child's blood as the first detector to the housing as the first detector, is the practical content of primary prevention in housing‐based lead policy. The map does not perform the home assessment or the remediation, and it does not retire blood‐lead testing, which remains necessary both to find children already exposed and to validate predictions like this one. The narrower claim is the one we would defend: where housing data flag a likely hazard in advance, the home can be tested before the child is. Used as intended, the map points limited inspection and abatement capacity at the housing where exposure is most likely, so a hazard can be found and controlled before a blood‐lead result would otherwise have revealed it.

A validated neighborhood risk map has a clear public‐health use. Childhood blood‐lead testing in the United States is targeted rather than universal, and the targeting is uneven. A current map of where exposure is most likely helps health departments and clinicians decide where to direct testing, outreach, and home‐hazard assessment first. Because the map covers every neighborhood from public data, it extends that guidance to the many areas that publish no measured surveillance of their own.

The map is most useful to the people and programs that already reach families with young children. Pediatric and prenatal care providers, Women, Infants, and Children (WIC) nutrition clinics, Medicaid managed‐care plans and their Early and Periodic Screening, Diagnostic, and Treatment programs, home‐visiting programs, Head Start, and childhood lead poisoning prevention programs all serve the at‐risk population and all decide whom to test, counsel, and refer. A free, neighborhood‐resolution risk layer lets any of them flag which of the families they already serve live in higher‐risk areas, and prioritize blood‐lead screening, anticipatory guidance, and home‐hazard education accordingly. Because the score is a property of place rather than of person, a provider can apply it to its own patient roster locally, without transmitting any patient information, which keeps the use within existing privacy practice. The same property makes the map a direct input to the geographic targeting plans the CDC asks state and local programs to maintain.

The neighborhoods this map ranks highest are disproportionately low‐income, and the residual analysis above indicates the map if anything under‐ranks the hardest‐hit Black and historically disinvested neighborhoods relative to their measured burden, the communities the documented racial ecology of lead exposure identifies as carrying it most unequally (Sampson & Winter, 2016). That distribution is part of why a free, universal tool matters. Families in these neighborhoods are among the least likely to live in a state that publishes neighborhood blood‐lead data, and the most likely to be reached only after a child's elevated result has already recorded the harm. A prediction available for every neighborhood lets a program direct limited prevention and remediation capacity to them first, before exposure rather than after. That is the equity case alongside the public‐health one: the burden falls hardest on the communities least equipped to find it in advance, and a tool that costs nothing and covers every neighborhood is one way to narrow that gap.

The map ranks risk; it does not confirm a hazard in any specific home. It is the first pass. A physical test of paint, dust, or water in the flagged home is what establishes whether a hazard is actually present, and pairing the two directs limited inspection and abatement capacity to where a hazard is most likely. The cost‐effectiveness of any particular screening program is beyond the scope of this paper. It depends on local testing costs, follow‐through rates, and remediation practices we do not model here.

A more rigorous validation is available to the agencies that hold complete blood‐lead records. Our test used only publicly released, aggregate surveillance, which bounds how finely an outside party can check the map. State childhood lead poisoning prevention programs, Medicaid blood‐lead testing files, and the CDC's full surveillance hold individual, address‐level results for far more children than any public extract. Any of these custodians, or a research partner under an appropriate data‐use agreement, could validate and recalibrate the map against their complete registry at the address level, including in states we could not test here, while keeping the identifiable data inside their own systems. The map and its open pipeline are built to enable that validation, and we invite it.

### 3.2 Limitations and Ethics

This map is a screening tool. It predicts where childhood lead‐exposure *risk* concentrates, using public housing and poverty data, and we validate that prediction against measured childhood blood lead in 11 states across the tract, ZIP, and county scales: four independent tract‐scale states, five independent county‐scale states, two ZIP‐scale states, and two tract‐scale consistency checks against the source study's own surveillance (several states are measured at more than one scale). It does not measure lead in any home, and it does not diagnose any child. Each limitation below constrains a specific claim a reader might otherwise draw from a colored map.

### 3.3 The Estimates Are Ecological

Every value on this map is a property of a census tract, not of a person or a house. The model is built from tract‐level aggregates (three construction‐era housing‐age shares, pre‐1940, 1940–1959, and 1960–1979, from Census table B25034, weighted toward older stock, and tract poverty from table S1701), so its output describes the average risk environment of a neighborhood. Reading a tract‐level association as if it applied to an individual is the ecological fallacy (Subramanian et al., 2009). The EPA hotspots analysis we extend is explicit on this point. Its authors state that the analysis operates at the population level (census tract, county, and state) and “cannot identify sources at particular addresses or risk at an individual level” (Zartarian et al., 2024). A high‐risk tract still contains remediated and lead‐free homes. A low‐risk tract still contains pre‐1940 houses with deteriorating lead paint. The map narrows where to look, but it cannot tell any single family whether their home has a hazard. That is what a physical test is for.

### 3.4 Scoring the Housing Itself

The index is computed for every tract regardless of how many young children live there. For a secondary‐prevention use, deciding where to send blood‐lead tests to find children already exposed, that is a limitation: predicted risk is essentially uncorrelated with the under‐five share (Spearman 0.065), so the ranking neither concentrates on nor avoids tracts with young children, and about 2.1% of scored tracts (1,754 of the 83,373 scored tracts that carry a Census child‐age record; 15 of the 83,388 scored tracts lack one and drop from this join) hold fewer than 10 children under five (under‐five counts from ACS 2018–2022 table B01001, fields B01001_003E and B01001_027E over B01001_001E; reproduced by child_presence.py, with output in data/child_presence.json); a screening program should overlay young‐child counts to weight outreach by the children actually present. For the primary‐prevention use this paper argues for, ranking the housing itself, current child presence is not the right filter. Lead paint in old housing is a persistent hazard. A tract with few children today still holds the same deteriorating paint, and as households turn over a child will eventually live in it and be exposed unless the hazard is controlled first. A system that waits for a resident child to register an elevated blood‐lead result guarantees that some child is exposed before anything is done; ranking and remediating the housing ahead of that exposure is the point. The child‐presence overlay sharpens the screening use, it does not bound the prevention use.

### 3.5 What the Map Predicts

The indices are correlates of exposure risk (old paint, concentrated poverty) rather than a measurement of lead in a child's blood. Our validation shows that the predicted surface tracks measured childhood blood lead at a moderate level (Spearman rho 0.54 in Michigan, 0.62 in Ohio, and 0.64 across Wisconsin statewide). A correlation of that order is meaningful for an ecological model and weak as a basis for any individual prediction. Much variance remains unexplained, because exposure also depends on factors absent from the model: actual paint condition, renovation and disturbance history, water service‐line material, soil, imported consumer goods, and occupational take‐home lead. The map should be read as a way to set priorities, never as a count of poisoned children.

### 3.6 The Input Data Carry a Vintage Lag

The map is only as current as the American Community Survey behind it. The 2018–2022 ACS 5‐year estimates pool responses collected across the full five‐year window of 1 January 2018 through 31 December 2022, so the housing and poverty picture is a multi‐year average rather than a snapshot of today (U.S. Census Bureau, 2023a). Housing stock changes slowly, which makes the pre‐1940 share relatively stable, but poverty, occupancy, and demolition or renovation can shift faster than the data refresh. Any tract that has seen significant teardown, rehabilitation, or demographic turnover since the survey window will be characterized with a lag of several years. The EPA analysis faced the same constraint. That work relied on 2010 census inputs. We improve currency by moving to the 2018–2022 vintage, but we do not eliminate the lag (Zartarian et al., 2024).

### 3.7 Small and Rural Tracts Are Measured Least Precisely

ACS reliability degrades as geography shrinks, and the 5‐year tract estimates that this map depends on are the Census Bureau's least precise published level. The Bureau ships a margin of error with every estimate for exactly this reason and urges caution where it is large (U.S. Census Bureau, 2018). The problem is built into the sampling. In the 2007–2011 ACS the average tract had only about 135 completed interviews over five years, against an average of about 280 housing units in the 2000 long form, and tract‐level margins of error run on average about 75% larger than the corresponding 2000 long‐form figures (Spielman et al., 2014). The 5‐year estimates that absorbed the COVID‐disrupted 2020 collection year carry wider margins still (U.S. Census Bureau, 2022). The consequence falls hardest on low‐population and rural tracts, where small samples widen the uncertainty band and a tract's percentile rank can be noisy. Sparse rural areas can also be physically large, so a single risk value may average over heterogeneous housing across many miles. Read rural risk on this map with more caution than urban risk, not less.

### 3.8 Validation Correlations Are Spatially Autocorrelated

The tracts and counties used to validate the map are not independent observations. Neighboring areas resemble one another in both predicted risk and measured blood lead, so the effective number of independent units is smaller than the raw count, and an ordinary bootstrap that ignores this reports intervals that are too narrow. We address it at the census‐tract scale with a spatial block bootstrap that resamples whole geographic blocks, which widens the intervals to reflect the clustering, and we report Moran's I of the residual to show how much spatial structure remains (it is substantial, 0.49 to 0.56 at the tract scale, all permutation *p* near 0.001). Because autocorrelation this large persists after blocking, the effective number of independent tracts is well below the raw count, and the width of a block bootstrap interval depends on the block scale chosen (we set the grid at about the fourth root of the tract count, targeting blocks of roughly 30 to 50 tracts; a completely separate county‐block resample, a different blocking unit, lands on the same intervals, which is the main evidence the grid choice is not driving the width), so the tract‐scale intervals should be read as approximate rather than exact. Wisconsin is now measured statewide across 71 counties, so its interval is corroborated by a county‐block resample (0.45–0.75) as well as by the grid blocks, the way Michigan's and Ohio's are. At the county scale the resampling unit is already the county, but Missouri and New York still carry residual spatial autocorrelation, so their intervals are optimistic and we report those two as point estimates outside the headline county range. None of this moves the point estimates; it widens the uncertainty around them, and it is why we do not lean on any single state. The point estimate does carry the broad regional trend the autocorrelation reflects, which is why we also report the within‐county decomposition: with county means removed, the Wisconsin correlation is still 0.50, a check that the ranking holds between neighborhoods of the same county and not only across regions.

### 3.9 The Analysis Was Not Preregistered

The weighting was fixed in advance and not fitted to the blood‐lead data, and the weight‐sensitivity analysis shows the result does not turn on it, but the study was not preregistered and several choices were made with the data in view. The inclusion rule that keeps a county series only when its interval clears zero at the 3.5 μg threshold was applied after the correlations were computed; it is what excludes the Massachusetts county series. We report every series we computed, including the ones the rule excludes (the Massachusetts county result at rho 0.42, and Missouri and New York with their optimistic intervals), so the rule subtracts nothing from the record and only decides what enters the single headline county range. We report 11 states across several testing‐volume floors and nine weighting schemes without a formal correction for multiple comparisons. The headline rests on effects that are individually well clear of zero and consistent across independent states and scales rather than on any single borderline test, and the results should be read as an exploratory, descriptive body of evidence whose weight rests on the consistency of sign and magnitude across many independent surveillance systems and scales, not as a set of confirmatory hypothesis tests.

### 3.10 Measured Blood‐Lead Data Are Scarce and Uneven, Which Is Why Prediction Is Needed

The reason a prediction map is needed is that ground‐truth blood‐lead data are incomplete and inconsistently available. Surveillance undercounts exposure because not all children are tested. CDC receives about 3 million blood‐lead test results a year, and the agency states plainly that these data are “not a population‐based estimate” and “are not representative of the United States or even of an entire state or county” (CDC, n.d.‐c). We test directly whether this targeting drives the result and find it does not: the measured values are rates, not counts, and controlling for the number of children tested leaves the correlations positive (Sensitivity to testing coverage, above), though a validation fully independent of who gets tested would need population‐representative testing or a complete registry. Coverage and reporting vary by state, by insurer, and by year. That scarcity has two consequences for this work. First, our validation rests on the states that publish tract‐ or ZIP‐resolvable measured data (Michigan and Ohio via the EPA paper's Supplement B; Wisconsin statewide, plus Minnesota and Rhode Island via official state health‐department ArcGIS services and Massachusetts via a Harvard Center for Geographic Analysis data set built on MA DPH surveillance; New York and Missouri by ZIP), so external validity is supported across a range of housing eras and testing regimes within these regions, though every validation state is an older‐housing Northeast, Midwest, or Great Plains jurisdiction and not every region can be checked, a limitation we state next. Second, the same scarcity is the public‐health case for the map. It extends a neighborhood‐level warning to the many jurisdictions (e.g., New Hampshire, Colorado, and Connecticut) where measured blood‐lead data are locked in PDFs or Tableau dashboards and reachable only by FOIA. Where there is no public blood data, a validated prediction is the only neighborhood‐level signal available.

### 3.11 The Validation States Are All Older‐Housing Regions

All 11 validation states (Michigan, Ohio, Wisconsin, Massachusetts, Minnesota, Rhode Island, New Jersey, Illinois, Missouri, Iowa, and New York) sit in the Northeast, Midwest, and Great Plains, the older‐housing regions Figure 1 scores highest. None is a newer‐built Western, Mountain, Pacific, or Sun Belt state. The map's low‐risk predictions, and its accuracy where non‐paint pathways such as soil lead, lead service lines, and imported consumer goods loom larger than old paint, are therefore validated nowhere. This is a matter of where measured data are published, not a gap we declined to fill. At the 3.5 μg‐per‐deciliter classification and 2022 vintage used for our county validation, the CDC Tracking Network carries no usable county values for the newer‐built West and Sun Belt: California and Utah return no county series, Arizona, Nevada, and Texas return county rows with every value suppressed, and Florida and Georgia are populated for only a sparse subset of their largest counties (19 of 67 and 22 of 159 counties, respectively). The measured ground truth needed to check the map's low predictions there is not published at usable resolution, which is the data‐side reason those predictions remain an explicit, untested extrapolation. We show the ranking holds where housing age varies within the older‐housing regions; we do not show that it transports to the West and South, and that transportability is a hypothesis the present data cannot confirm.

### 3.12 The Line Between Screening and Diagnosis

The right framing for both the map and the test it points to is screening. CDC is explicit that even its blood‐lead reference value of 3.5 μg per deciliter is “a screening tool,” is “not health‐based,” and is “not a regulatory standard,” and that no safe level of lead in children has been identified (CDC, 2021a). A neighborhood risk map sits one step further from diagnosis than a blood test does. It flags places to investigate, and nothing more. It does not establish that a hazard exists at any address or that any child has been exposed. The right response to a high‐risk tract is confirmation, not alarm. That means a home risk assessment, a dust‐wipe or paint‐chip analysis, or an XRF reading, followed by a blood‐lead test for the child if a hazard is found. Used that way, the map does what a screen should do. It points limited inspection and testing resources toward the places most likely to need them, and it makes no claim it cannot support about any individual home or child.

## Conflict of Interest

The author is the founder of Fluoro‐Spec Inc. (DetectLead.com), which manufactures and sells a consumer lead‐screening product, and owns Spirochete Research Labs, LLC, which has interests in lead‐detection technology. The relevant conflict is direct and worth naming plainly: this paper argues that childhood lead screening is under‐targeted and should be expanded, and that high‐risk neighborhoods warrant in‐home confirmation, a policy from which the author's product could benefit commercially. The mitigations are that the prediction method and its validation are not original to the author (they extend the EPA and Washington State Department of Health approach and are reproducible from public inputs by any party), that the recommended confirmation step is generic and names no specific product (a certified risk assessment, an XRF reading, a dust‐wipe or paint‐chip analysis, or a blood‐lead test), and that the case for expanded screening rests on CDC and EPA positions rather than on the author's work. The map and method are built entirely from public data and provided free, with no login or paywall, which is a secondary mitigation rather than the rebuttal. The interactive map is hosted on the author's commercial domain; the analysis code and derived data that reproduce every reported result are archived independently at Zenodo, so no reproduction step depends on that site. Rather than assert that the commercial interest did not influence the work, which the author cannot prove, the stronger guarantee is reproducibility: the choice of data sources, the validation design, and every reported result can be re‐derived independently from the public inputs and the archived code, without relying on the author's word.

## Data Availability

All inputs are public. The risk model uses U.S. Census American Community Survey 2018–2022 five‐year tables (B25034 and S1701, and for the residual analysis B25077, B19013, B25003, B25002, B02001, B03002, and B17001) retrieved through the public Census API. Validation uses publicly released state and federal childhood blood‐lead surveillance: the CDC National Environmental Public Health Tracking Network county series, the Michigan and Ohio tract‐level surveillance, the Wisconsin DHS statewide open ArcGIS service, New York State open health data, and additional services for Minnesota, Rhode Island, and Missouri (official state health departments) and Massachusetts (Harvard Center for Geographic Analysis, from MA DPH surveillance), all reproduced by additional_states.py. The analysis code, the derived tract‐ and county‐level risk scores, and the state validation joins are archived at Zenodo under a CC‐BY‐4.0 license (Ritter, 2026); the archive's concept DOI resolves to the latest archived version. The interactive national map is at detectlead.com/lead‐risk‐map. Because the derived risk scores and the validation joins are archived alongside the code, the headline validation figures (the per‐state correlations, the screening‐yield figures, and Figure 2) rebuild from the archive without re‐pulling any live service. The residual, weight‐sensitivity, and benchmark analyses re‐pull the per‐tract ACS predictor variables from the free, keyed Census API, because those variables are not redistributed in the deposit; the live ArcGIS and Socrata pulls likewise refresh the measured data but are not required to reproduce the headline results. No individual‐level, identifiable, or access‐restricted data were used.
